# A post-market, multi-vessel evaluation of the imaging of peripheral arteries for diagnostic purposeS comparing optical Coherence tomogrApy and iNtravascular ultrasound imaging (SCAN)

**DOI:** 10.1186/s12880-020-0420-7

**Published:** 2020-02-14

**Authors:** Edward Pavillard, Luke Sewall

**Affiliations:** 1 Pennsylvania Vascular Institute, 420 W. Linfield-Trappe Road Suite 3200, Limerick, PA 19468 USA; 2 AMITA Health, 911 N. Elm Street Suite 128, Hinsdale, IL 60521 USA

**Keywords:** Intravascular imaging, IVUS, OCT, Plaque, Diagnosis, Treatment strategy

## Abstract

**Background:**

Intravascular imaging plays an important part in diagnosis of vascular conditions and providing insight for treatment strategy. Two main imaging modalities are intravascular ultrasound (IVUS) and optical coherence tomography (OCT). The objective of this study was to prove non-inferiority of OCT imaging to IVUS images in matched segments of peripheral vessels in patients with suspected peripheral vascular disease.

**Methods:**

The SCAN study was a prospective, non-inferiority clinical study of matched IVUS and OCT images collected along defined segments of peripheral vessels from twelve subjects (mean age 68 ± 10.3 years; 10 men) displaying symptoms of vascular disease. Luminal diameters were measured by both imaging systems at the distal, middle, and proximal points of the defined segments. Three blinded interventional radiologists evaluated the quality of both imaging modalities in identifying layered structures (3-point grading), plaque (5-point grading), calcification (5-point grading), stent structure (3-point grading), and artifacts (3-point grading) from 240 randomly ordered images. Mean grading scores and luminal diameters were calculated and analyzed with Student’s t-Test and Mann-Whitney-Wilcoxon testing. Intrareader reproducibility was calculated by intraclass correlation (ICC) analysis.

**Results:**

The mean scoring of plaque, calcification, and vascular stent struts by the three readers was significant better in terms of image quality for OCT than IVUS (*p* < 0.001, *p* = 0.001, *p* = 0.004, respectively). The mean scores of vessel wall component visibility and artifacts generated by the two imaging systems were not significantly different (*p* = 0.19, *p* = 0.07, respectively). Mean vessel luminal diameter and area at three specific locations within the vessels were not significantly different between the two imaging modalities. No patient injury, adverse effect or device malfunction were noted during the study.

**Conclusions:**

Imaging by OCT provides the physician with better visualization of some vessel and plaque chacteristics, but both IVUS and OCT imaging are safe and effective methods of examining peripheral vessels in order to perform diagnostic assessment of peripheral vessels and provide information necessary for the treatment strategy of peripheral artery disease.

**Trial registration:**

NCT03480685 registered on 29 March 2018.

## Background

Intravascular imaging has been used for many years in visualization and characterization of coronary vessel morphology and presence of atherosclerotic plaque, resulting in improved success in treatments and clinical outcome due to better risk stratification [1, 2]. For example, the evaluation of stent position and appropriate sizing of coronary stents by OCT imaging can determine stent malposition so that further dilation can be accomplished to improve stent placement at the time of the procedure [3].

Drawing on the utility in coronary vessels, intravascular imaging has become much more frequently utilized as an adjunct to angiography in the treatment of peripheral artery disease (PAD). The ability to visualize internal vessel architecture provides clinicians with information useful in the evaluation of stenosis, dissection and plaque morphology. Intravascular imaging can therefore assist in the development or modification of a treatment strategies [4]. Such imaging has also shown utility in post-treatment assessments, which can result in increased treatment success and a reduction in patient morbidity [5, 6].

The two primary modalities of such imaging are intravascular ultrasound (IVUS) and optical coherence tomography (OCT). As the name denotes, IVUS interprets high-frequency sound waves that rebound off vessel walls and are collected by a processing system. The intensity of the sound waves varies depending on the tissue encountered and the operating system processes the signals in order to create a cross-sectional image [7]. IVUS can be used to measure plaque extent, morphology and distribution, but it has low spatial resolution (150 μm) and calcium deposits in the vessel walls can reduce penetration of the sound waves [8, 9]. In contrast, OCT imaging measures the intensity reflected near-infrared light that is captured by a system to develop images of tissue and structures [10]. OCT images have higher resolution (10 μm) and faster imaging acquisition than IVUS [11], but such imaging requires management of blood flow that can interfere with light transmission [12].

**Fig. 1:**
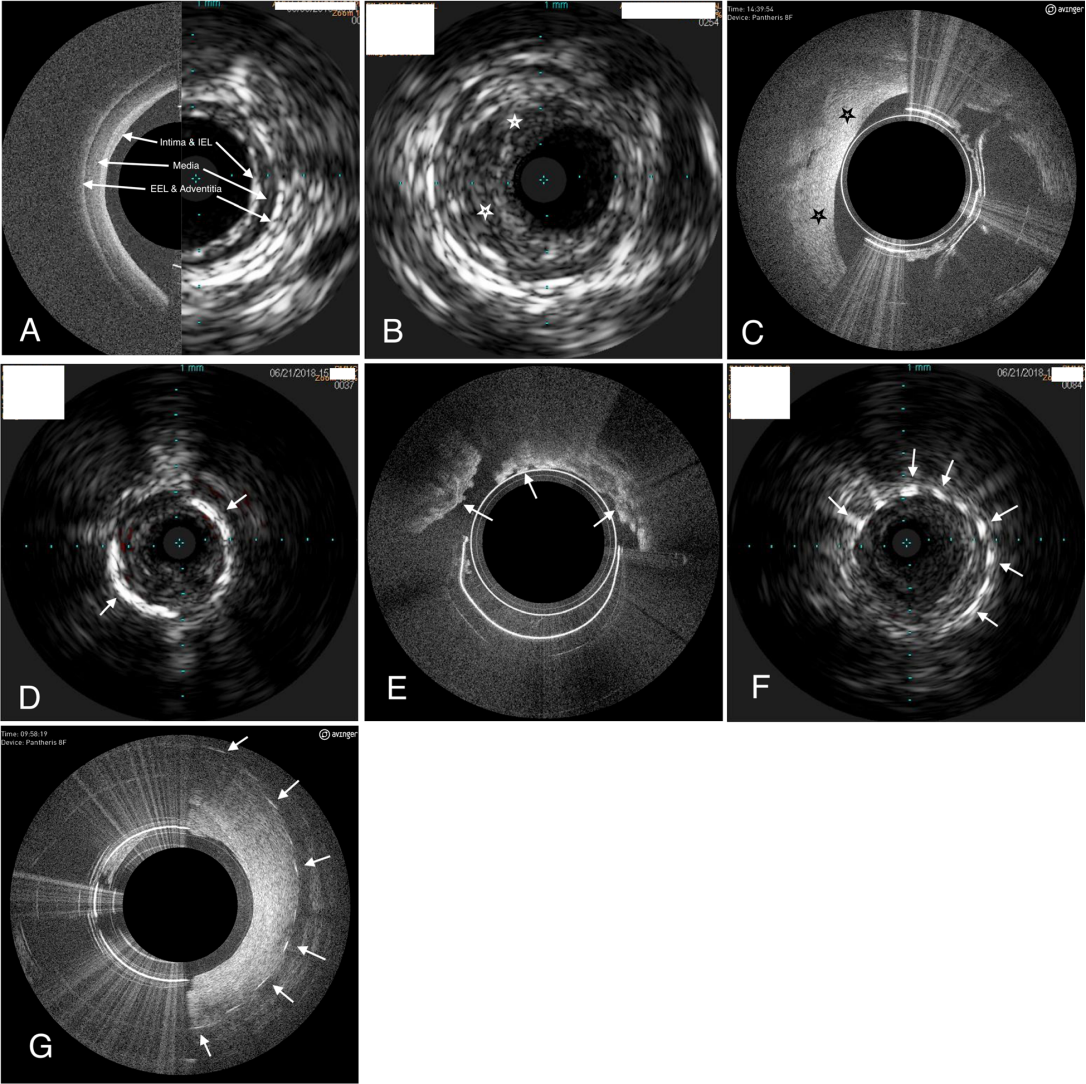
Morphologies and characteristics of peripheral vessels noted with either IVUS or OCT imaging. **a** Layered structures of the vessel wall shown with OCT (left) and IVUS (right) imaging. IEL – internal elastic lamina; EEL – external elastic lamina. **b** Plaque (stars) present in the vessel wall denoted by IVUS imaging. **c** Plaque (stars) present in the vessel wall presented by OCT imaging. **d** Calcium present in the vessel imaged by IVUS (arrows). **e** Calcium as imaged by OCT (arrows). **f** Vascular stent struts noted with IVUS (arrows). **g** OCT imaging of vascular stent struts (arrows)

IVUS and OCT imaging in peripheral vessels has been noted to have comparable utilities in peripheral vessels to their use in coronary arteries through assessing vessel characteristics and morphology, such as vessel and lumen diameter, area of stenosis, and plaque location and extent (Fig. 1) [13, 14]. By utilizing intravascular visualization, the physician is able to diagnosis the patient’s specific vascular condition and develop a treatment strategy to a level of refinement not possible with angiography [15]. The only previous comparative study of IVUS and OCT imaging in peripheral arteries was conducted with an OCT imaging catheter indicated for use in coronary vessels consisting in subjects with short lesions, low level of ischemia, and without thrombosis [11], all conditions that do not reflect the majority of patients presenting to peripheral vascular interventionalists for treatment. The purpose of this study was to prove non-inferiority of OCT imaging with a device indicated for peripheral vessels to IVUS images of the same segment of peripheral arteries of a clinically relevant patient population in order to assess real-world presentation of peripheral artery disease with the two imaging modalities.

## Methods

The evaluation of the imaging of peripheral arteries for diagnostic purpose**S** comparing optical **C**oherence tomogr**A**phy and i**N**travascular ultrasound imaging (SCAN) study was a two-center, prospective, non-inferiority study comparing the quality of IVUS and OCT imaging in the assessment of vessel characteristics for diagnosis and to support treatment strategy. The protocol was reviewed and approved by an institutional review board. All subjects signed an informed consent document prior to enrollment. The informed consent document provided to subjects followed guidelines outlined by Good Clinical Practices (GCP), the Declaration of Helsinki, and the International Conference on Harmonization (ICH). The study was registered on the National Institutes of Health website ClinicalTrials.gov (NCT03480685).

Subjects included in this study were adults (≥18 years old) with symptomatic peripheral arterial disease (Rutherford Class 2 or greater) that were scheduled for revascularization of a diseased vessel. Prior to any intervention, the subject was included if angiography determined that the reference vessel was of sufficient size to accommodate either imaging device and contained sufficient volume of plaque to treat with either atherectomy or other method of revascularization. Subjects were excluded only if female and pregnant or breast feeding or unwilling to give informed consent in order to provide plaque and other anatomic characteristics that reflect real-world cases. The procedures were performed on patients with common co-morbidities without acute hemodynamic instability.

Based upon the inclusion/exclusion criteria, twelve (12) subjects diagnosed with peripheral arterial disease of the lower extremities were enrolled, with the goal to acquire at least 120 matched images for analysis. With a sample size of 120 images by both the OCT and IVUS catheters, a two-group 0.05 one-sided matched *t*-test will have 90% power to reject the null hypothesis that the OCT and IVUS imaging modalities are not equivalent in favor of the alternative hypothesis that the two are equivalent.

### Imaging systems used

All OCT images were captured with the Pantheris catheter (Avinger, Inc.) and all IVUS images were captured with the Visions PV 0.014P catheter (Royal Philips Corp.). Both devices have marketing clearance by the United States’ Food and Drug Administration (FDA) for use in diagnostic imaging within peripheral vessels. Neither device is considered investigational nor experimental for this indication and, in this study, both were used in accordance with their FDA- cleared indications for use.

The Pantheris catheter is a monorail 7 French device with a working length of 110 cm. It contains a 155 μm optical laser fiber on the shaft of the catheter utilizing frequency domain for image capture. The catheter is connected to a console that displays the images. The device was designed for atherectomy in peripheral vessels, but the OCT-imaging capability of the catheter allows the physician to assess the burden and location of plaque within a vessel, as well as vessel morphology, prior to tissue excision so that the physician can diagnose the patient’s condition and plan the treatment strategy. In addition, the imaging allows the physician to monitor tissue excision in order to avoid healthy tissue and then, using measuring tools in the system’s software, measure vessel lumen diameter to determine appropriate sizing for a balloon or stent to be used in adjunctive treatments. Blood management is accomplished by inflation of a balloon proximal to the laser fiber. The catheter is provided sterile and is for single use only.

The Visions PV 0.014P IVUS catheter (Visions catheter) has an outer diameter of 5 Fr and a working length of 150 cm. It also is advanced through an indwelling vascular sheath following an 0.014″ guide wire. The catheter is connected with an imaging system that displays the vascular images. The IVUS catheter permits the clinician to assess disease markers, such as plaque burden and lesion location and morphology, as well as provide measurements of the lumen of the vessel. It is provided sterile and for single use only.

### Image acquisition

After providing informed consent, the subject was prepared for the diagnostic procedure according to the institution’s and investigator’s standard procedures. Demographic information was recorded for each subject and a radiopaque ruler (Glow N′ Tell, LeMaitre Vascular, Inc. Burlington, MA) was applied to the subject’s leg to provide reference marks for the starting and stopping points of the “target segment” to be imaged by the two devices. The ruler also provided reference points for the angiography cine.

Percutaneous access of the contralateral common femoral artery was obtained and a 7-French vascular sheath inserted up-and-over the aortic bifurcation and to the region of interest of the artery under fluoroscopic guidance. Next an 0.014 in. guide wire was advanced via the lumen of the sheath until its tip was distal to the target segment.

**Fig. 2:**
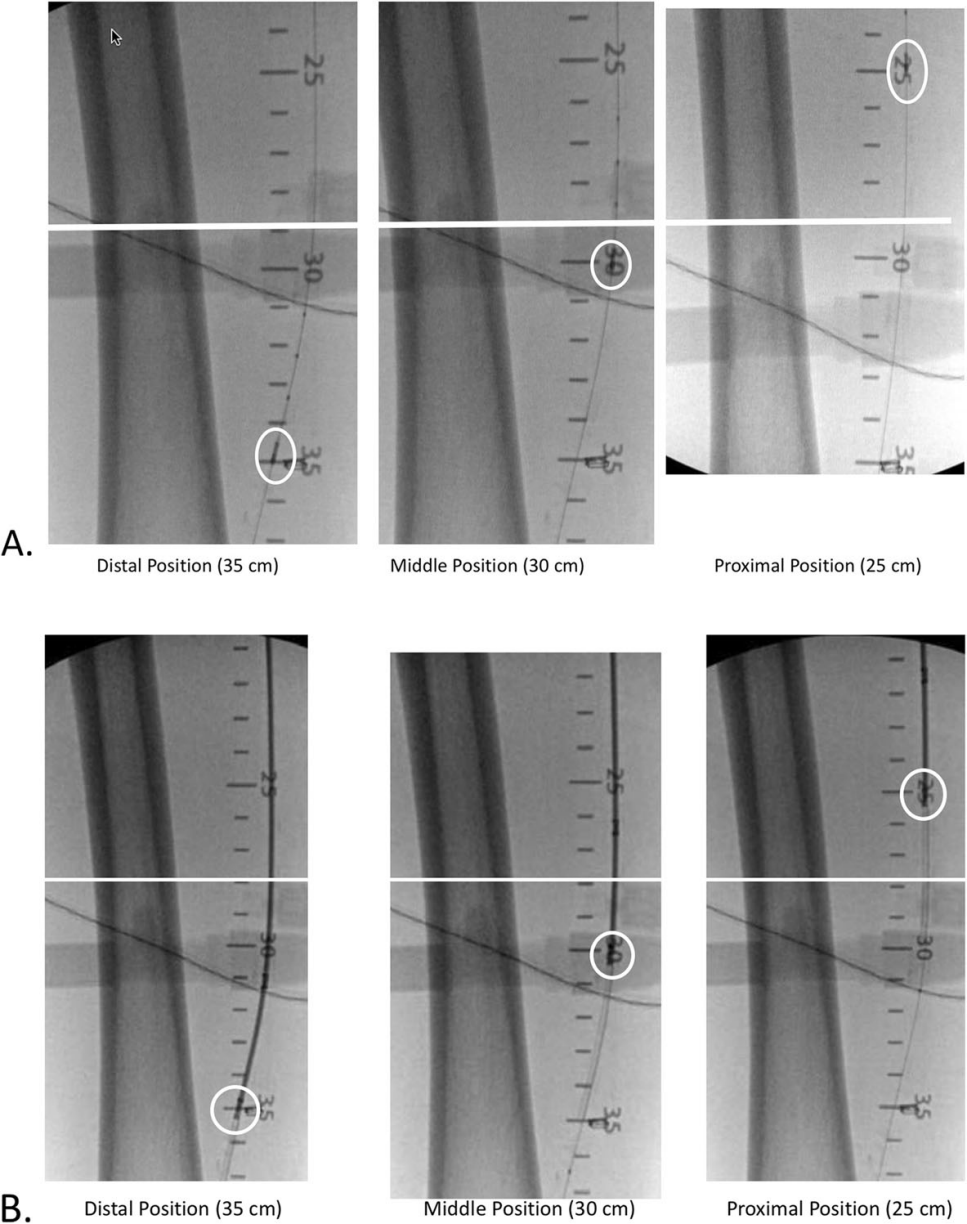
Example of distal, middle, and proximal image sampling points for (**a**) IVUS and (**b**) OCT imaging catheters

All images captured for review were contained within areas of interest in vessel segments containing disease or anatomical abnormalities within the markings of the radiopaque ruler. First an IVUS catheter was loaded onto the wire and advanced until its imaging transducer resided at the distal end of the target region (the starting point). Using the radiopaque ruler as a reference of the starting point (distal end) and stopping point (proximal end) of the target region, the IVUS catheter was energized for imaging and retracted by the physician through the target segment capturing images within that segment; no pull-back device was used. Following this imaging run, the IVUS catheter was again advanced to the distal point of the area of interest and a snapshot image of the vessel taken (Fig. 2), not only as a known point for comparison but also for measurement of the luminal diameter and area. Next the IVUS catheter was retracted to the mid-point of the target segment, which was within the region of disease, and another snapshot taken, followed by withdraw to the proximal point of the run for the final snapshot. Using the software of the IVUS console, shortest and longest diameter and area of the lumen at these three points were measured and recorded. After all images were captured on the IVUS system, the IVUS catheter was removed.

Next, using the same radiopaque ruler markings as a reference, the OCT-imaging catheter was advanced over the 0.014 guide wire until its imaging window was at the same starting point (distal end) of the target segment that had been used by the IVUS catheter. With the device energized, the OCT catheter was retracted through the exact same target segment of the vessel and the OCT images captured on the Lightbox system on a cine loop. As was performed with the IVUS catheter, the OCT catheter then was re-advanced to the distal point of the lesion or area of interest and a snapshot taken of the vessel, followed by subsequent snapshots at the middle and proximal points of the lesion. Vessel diameter and luminal area were determined from these snapshots using the measuring software of the Pantheris system. When all imaging with the IVUS and OCT catheters was complete, the subject exited the study.

Data procurement added less than 5 min to the expected procedural time and there were no adverse events as a result of capture of these images. The rate of manual pull-back of the OCT-imaging catheter averaged 23 s (SD 2.7 s) for one investigator and 27 s (SD 1.9 s) for the other. Over the same distances (proximal to distal capture endpoints), the IVUS catheters captured a mean of 1239 images (SD 298 images).

The IVUS images were downloaded from the system onto a DVD and transferred to a computer hard-drive for processing. The OCT images were captured on the Lightbox and downloaded to a memory stick prior to transfer to a computer hard-drive. The fluoroscopic images taken during each case were downloaded to a DVD by the hospitals’ radiology departments.

### Study endpoints

The primary efficacy of imaging endpoint was met when OCT imaging had the equivalent or higher ranking of IVUS imaging of the visualization of vessel morphology and disease for:

- Structure of the vessel wall – intima, media, external elastic lamina, and adventitia;
- Non-layered structure—presence of disease (plaque) in the vessel structure;
- Abnormal physiology— presence of calcification; and
- Obscuring of the image by artifacts.

The primary safety endpoints were freedom from diagnostic imaging procedure-related and device-associated adverse events at the time of the imaging, as reported by the physician.

Subject participation lasted the duration of the diagnostic imaging procedure. No follow-up images were collected after the procedure.

### Image analysis

The IVUS and OCT images were reviewed for suitability of use in the study by a consultant with over twenty years of experience in the interpretation of angiography and IVUS and OCT images of peripheral arteries. For each run of the target segments, the start, middle, and stop locations of the target segments in both the IVUS and OCT image files were identified from distinct points on the angiographic ruler and from the procedure notes. To gain additional images for review, IVUS and OCT images were matched to locations along the entire target segment to within 1 mm, using the fluoroscopic images as a reference. The imaging series resulted in over 1000 imaging pairs, but for each subject the matched images that best displayed the characteristic to be assessed were chosen for use in the study library.

**Fig. 3:**
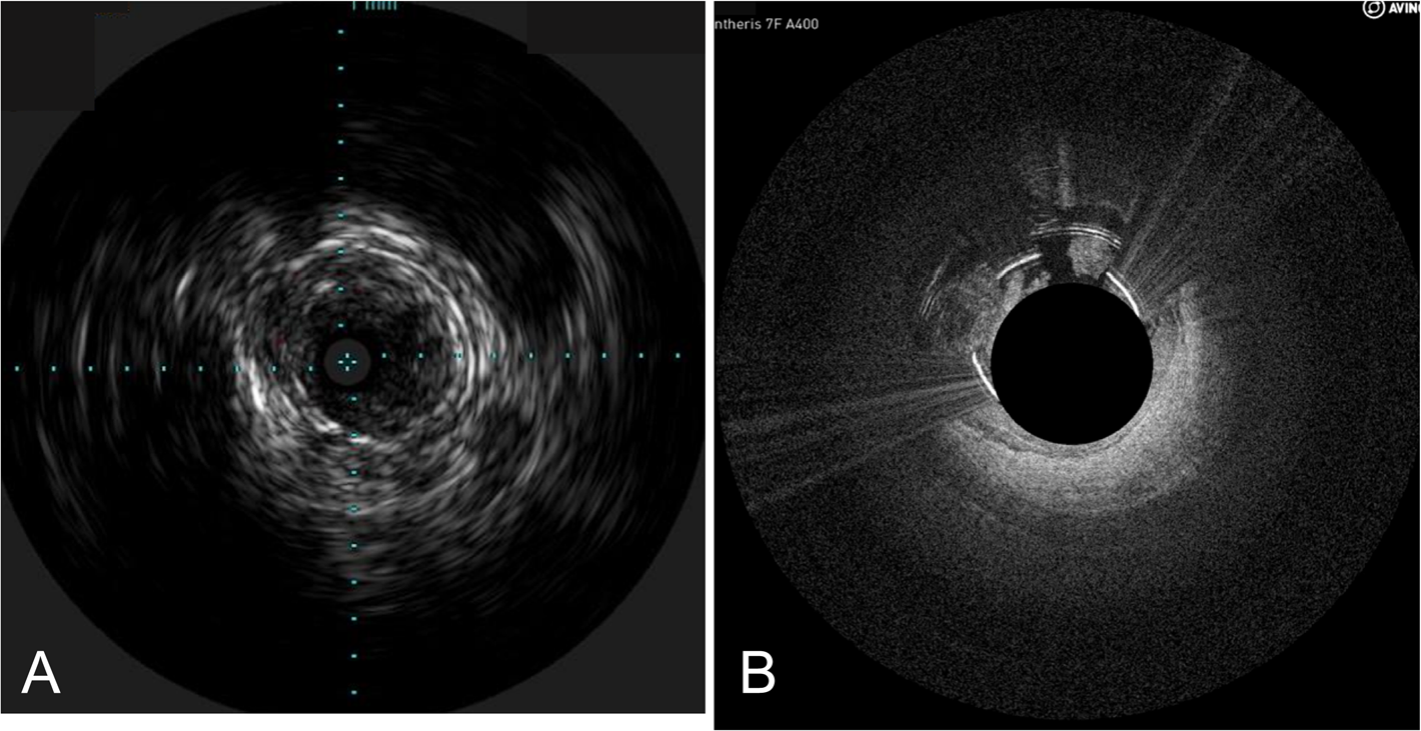
Matched images of the nonlayered structures at the same location in a peripheral artery from IVUS imaging (**a**) and OCT imaging (**b**). The IVUS image was displayed as number 228 in the series of images provided to the readers and the OCT image was displayed as number 14

The images were anonymized and given a unique identification number prior to assessment by the readers. Since the presentation of IVUS and OCT images are visibly different, the readers could not be blinded as to which imaging device was used to generate the image, but it was important that the images not be presented as matched images in sequence. Therefore, the images were provided in a random sequence so that the reader could not match a specific IVUS image to a specific OCT image at the time of the review. For example, Fig. 3 shows IVUS and OCT images from the same location in a peripheral artery of one subject in the study. The IVUS image was identified in the series of images as number 228 and the OCT image was identified as image number 14. In this example, after viewing the OCT image, the readers would have viewed 214 other images between seeing the IVUS image from that same subject at that same point in the vessel, which reduced the chance that the reader would recognize the two images as coming from the same vessel location. This resulted in 120 images matched to each imaging catheter for a total of 240 images to be reviewed. Within these 120 images, the characteristic to be ranked appeared either singly on the image or in conjunction with other characteristics, which resulted in 309 scores from each reader and a total of 927 scores (Table 1).

**Table 1:** Patient and vessel characteristics

No. of patients	12
Mean age, years (range)	68 (55–87)
Sex	Male 10 (83%). Female 2 (17%)
Race	Caucasian 92%. African-American (8%)
Mean weight, kg (range)	82 (69–92)
Mean height, cm (range)	176.8 (167.6–187.9)
Leg of the vessel accessed	Left 9 (75%). Right (25%)
Vessel imaged	SFA (64%) Popliteal (36%)
Mean length of target segment of vessel, cm (range)	24 (10–60)
Number of images containing a specific vessel characteristic to be scored	
Layered structure	24
Nonlayered structure	104
Calcification	40
Stent	21
Artifact	120

The images were reviewed by three independent readers, who were experienced interventional radiologists. The readers were not present during the cases at which the images were captured nor were they associated, professionally or otherwise, with the hospitals at which the images were collected. All of the readers had experience in the interpretation of IVUS and OCT images. Prior to the image assessment stage, representative images were reviewed with the readers in order to create a consistent nomenclature when identifying different tissue types with both imaging catheters and to standardize the image review process.

The images were provided in eight blocks of 30 images and the readers ranked each for clarity (using a visual analogue scale or VAS) in the diagnosis of vessel condition and presence of plaque pre-procedure. Qualitative interpretation of OCT and IVUS images were ranked using the convention of Eberhardt et al. [11] as follows:

- Layered structure – intima, media, external elastic lamina and adventitia (ranked as 1- clear differentiation of all the vessel wall layers, 2- differentiation of at least three vessel wall layers, 3- differentiation of at least two vessel wall layers, and 4-no differentiation of any vessel wall layer);
- Non-layered structure —presence of plaque in the vessel structure (1- excellent histology-like image quality to 5- unacceptably poor image quality);
- Abnormal physiology—calcification (1- excellent histology-like image quality to 5- unacceptably poor image quality); and
- Effect of artifacts on reading the images (1- no artifacts present, 2- present and tolerable but not limiting diagnostic image quality, 3- intense and does limit diagnostic image quality).

The results of all readings of both imaging modalities were used for intrareader comparison to evaluate the reproducibility and reliability of the respective imaging modality.

### Statistical analyses

Statistical analyses were carried out using StatView software (SAS Institute, Cary, NC). A *p* value < 0.05 was considered statistically significant. Matched Pairs *t*-Test were performed to compare means and on paired samples to compare procedural data and complications associated with the imaging techniques. The Mann-Whitney-Wilcoxon test was applied for statistical comparison of the scores of all three readers. A two-way intraclass correlation (ICC) was calculated to assess intra-reader reproducibility.

### Safety reporting

Incidence and severity of procedure-related and device-related adverse events (e.g., vessel spasm, thrombosis, distal embolism, etc.) were evaluated following each scan and documented over the course of the study.

## Results

Demographics of the twelve subjects who met the inclusion/exclusion criteria are reported in Table 1. Images were taken primarily within superficial femoral arteries (SFA) of the left leg. The mean length of the selected vessel segments was 16 cm, ranging from 5 to 30 cm.

### Safety and catheter performance

Of the twelve subjects in the study in which both IVUS and OCT catheters were placed, no periprocedural or post-procedure adverse events occurred. Intraoperative monitoring of heart rate and blood pressure prior to insertion of and during image capture by each catheter had no clinically relevant fluctuations or deviations.

The time required for scans by either system was comparable (mean of 83 s for IVUS and a mean of 94 s for OCT), with no repeated vessel segment runs required in order to capture the images. All cases of imaging were performed with a single IVUS or OCT catheter, with no catheter malfunctions. Images were recorded fully on both systems and available for image extraction at the time needed.

### Qualitative image analysis

The OCT and IVUS images displayed comparable levels of clarity at the 3 specific points of the target vessel segments imaged. While the scores for IVUS imaging were positive for all vessel characteristics, indicating that the readers could distinguish vessel characteristics sufficiently, the OCT images scored statistically higher for the overall visual quality of the non-layered structures (plaque), calcification, and stent struts. The two imaging modalities were not significantly different in scoring of the layered structure or any artifacts obscuring the image. The location of specific images within the vessel segment imaged did not affect the rankings; although, the acceptability of the imaging to the three readers scored significantly different between the IVUS and OCT images (Table 3). The statistical difference in image quality between the two modalities is supported by rounding the mean scores in Table 2 to whole numbers or when viewed as the median scores for each element. The median score for layered structures was 1 for IVUS imaging and 1 for OCT imaging; for plaque differentiation the IVUS had a median score of 3 verusus a median of 2 for OCT images; calcium and artifacts had median scores of 2 for both imaging modalities, while the median score for stent structure recognition was 1 for OCT imaging verusus 2 for IVUS.

**Table 2:** Mean ranking of the image quality of layered structure, non-layered structure, calcification, stent structure, and artifacts as rated by three readers of matched images captured by OCT and IVUS systems

Vessel Characteristic	Mean score for IVUS images	Mean score for OCT images	Student’s *t*-Test	Mann-Whitney-Wilcoxon test
Layered structure^a^	1.61	1.49	*p = 0.19*	*z = 0.82* *p = 0.41*
Non-layered structure^b^	2.70	1.82	*p < 0.001*	*z = 11.04* *p < 0.001*
Calcification^c^	2.45	2.11	*p = 0.001*	*z = 2.80* *p = 0.005*
Stent structure^d^	1.79	1.43	*p = 0.004*	*z = 2.67* *p = 0.007*
Artifacts^e^	1.87	1.79	*p = 0.07*	*z = − 1.52* *p = 0.12*

Scoring: ^a^ Layered structure (1- clear differentiation of vessel wall layers, 2- differentiation of 3 wall layers, 3-differentiation of 2 wall layers, 4- no differentiation visible); ^b^ Non-layered structure (1- excellent histology-like image quality to 5- unacceptably poor image quality); ^c^ Calcification (1- excellent histology-like image quality to 5- unacceptably poor image quality); ^d^ Stent structure (1 excellent image, 2 – acceptable image, 3 – unacceptably poor image); and ^e^ Artifacts (1-none, 2- tolerable/not limiting, 3-is intense and limits image quality)

The intrareader reproducibility for the quality of images measured were over 0.70 for both OCT and IVUS images (Table 3).

**Table 3:** Mean ranking of the image quality of layered structure, non-layered structure, calcification, stent structure, and artifacts as rated by three readers of matched images captured by OCT and IVUS systems

Reader	Layered Structure^a^		Non-Layered Structure^b^		Calcification^c^		Stent Structure^d^		Artifacts^e^	
	IVUS	OCT	IVUS	OCT	IVUS	OCT	IVUS	OCT	IVUS	OCT
1	1.58	1.12	2.97	2.08	2.28	2.05	2.00	1.48	2.15	1.68
	*p = 0.002*		*p < 0.001*		*p < 0.001*		*p < 0.001*		*p < 0.001*	
ICC intrareader	0.82	0.91	0.89	0.91	0.77	0.83	0.82	0.93	0.85	0.81
2	1.42	1.04	2.55	1.54	2.15	1.60	1.81	1.14	1.73	1.51
	*p = 0.02*		*p < 0.001*		*p < 0.001*		*p < 0.001*		*p < 0.001*	
ICC intrareader	0.70	0.83	0.73	0.81	0.91	0.85	0.84	0.88	0.92	0.83
3	1.83	2.29	2.58	1.84	2.95	2.68	1.52	1.67	1.22	2.33
	*p = 0.005*		*p < 0.001*		*p = 0.19*		*p = 0.52*		*p < 0.001*	
ICC intrareader	0.84	0.75	0.89	0.77	0.92	0.87	0.83	0.86	0.92	0.81

Scoring: ^a^ Layered structure (1- clear differentiation of vessel wall layers, 2- differentiation of 3 wall layers, 3-differentiation of 2 wall layers, 4- no differentiation visible); ^b^ Non-layered structure (1- excellent histology-like image quality to 5- unacceptably poor image quality); ^c^ Calcification (1- excellent histology-like image quality to 5- unacceptably poor image quality); ^d^ Stent structure (1 excellent image, 2 – acceptable image, 3 – unacceptably poor image); and ^e^ Artifacts (1-none, 2- tolerable/not limiting, 3-is intense and limits image quality)

### Quantitative image analysis

Lumen diameters as measured with OCT and IVUS at distal, middle, and proximal points of the vessel target segments were comparable between the two imaging catheters in all measurements other than the shortest diameter in the middle of the targeted vessel segment (Table 4).

**Table 4:** The longest and shortest luminal diameter of vessels at the distal, middle, and proximal portions of the target segments of vessels and the resultant luminal area as measured by the Pantheris OCT or Visions PV IVUS systems

		Proximal Location			Middle Location			Distal Location		
Patient	Imaging Modality	Longest Diameter (mm)	Shortest Diameter (mm)	Luminal Area from Longest Diameter (sq mm)	Longest Diameter (mm)	Shortest Diameter (mm)	Luminal Area from Longest Diameter (sq mm)	Longest Diameter (mm)	Shortest Diameter (mm)	Luminal Area from Longest Diameter (sq mm)
P1	IVUS	5.4	4.3	22.9	5.1	4.6	20.43	3.5	3	9.62
	OCT	6.1	3.9	29.22	5.7	4.2	25.52	3.8	3.3	11.34
P2	IVUS	4.3	4	14.52	3.8	3.2	11.34	2.9	2.5	6.6
	OCT	3.9	3.3	11.95	4.2	3	13.85	3.2	3	8.04
P3	IVUS	3.9	3	11.95	4.4	4	15.2	3.1	2.6	7.54
	OCT	4.2	2.9	13.85	4.6	4.3	16.62	3.2	2.7	8.04
P4	IVUS	6.4	5.9	32.17	5.2	4.7	21.24	3.4	3.3	9.08
	OCT	5.7	5.3	25.52	4.8	4.4	18.09	3.1	2.9	7.55
P5	IVUS	6.7	5.8	35.26	5.9	5.1	27.34	5.9	5.4	27.34
	OCT	5.7	5	25.52	5.5	4.8	23.76	5.5	5.1	23.76
P6	IVUS	4.8	4.1	18.09	3.8	3.1	11.34	3.3	2.8	8.55
	OCT	5.4	4.1	22.9	3.6	2.8	10.18	3.5	3	9.62
P7	IVUS	5.3	4.3	22.06	4.6	4	16.62	3.2	2.8	8.04
	OCT	4.6	4	16.62	4.2	3.5	13.85	3	2.6	7.06
P8	IVUS	5.3	4.3	22.06	5.4	4.8	22.9	4.6	4.2	16.62
	OCT	5.6	4.7	24.63	5.1	4.3	20.43	4.9	4.3	18.86
S1	IVUS	3.5	3	9.62	3.6	2.5	10.18	3	2.3	7.07
	OCT	3.8	3.2	11.34	3.8	2.6	11.34	3.1	2.4	7.55
S2	IVUS	2.9	2	6.6	2.2	1.8	3.8	2.3	2.1	4.15
	OCT	2.8	2.5	6.16	2.4	2	4.52	3.2	2.4	8.04
S3	IVUS	3.7	2.9	10.75	3.9	3.4	11.94	3.3	2.7	8.55
	OCT	4.1	2.4	13.2	3.4	2.6	9.08	3	2.5	7.07
S4	IVUS	4.6	4.2	16.62	3.9	3.4	11.94	3.1	2.8	7.55
	OCT	4.3	3.9	14.52	3.7	3.1	10.75	2.8	2.5	6.16
MEANS	IVUS	4.7	3.9	18.55	4.3	3.7	15.36	3.5	3	10.06
	OCT	4.7	3.8	17.28	4.2	3.4	14.83	3.5	3	10.26
		*p = 0.80*	*p = 0.10*	*p = 0.39*	*p = 0.54*	*p = 0.02*	*p = 0.52*	*p = 0.60*	*p = 0.96*	*p = 0.74*

## Discussion

This is the first study comparing the quality of IVUS and OCT images in peripheral vessels in patients in the United States. The quality of the two modalities were comparable for differentiation of vessel wall layers and the amount of artifact present in the images, while OCT imaging was ranked as significantly better for imaging luminal plaque, calcium, and foreign body sturctures, such as stent struts. Even though some of the vessel characteristics were significantly better visualized by OCT than with IVUS, this study was designed to determine the characteristics of both imaging modalities to assist physicians in the diagnosis of and development of treatment strategy for patients with peripheral arterial disease. OCT imaging was comparable to imaging by IVUS technology for visualizing layered structures and having insufficient interference from artifacts to obscure the field of view, both of which provide important information to the physician during a procedure. While the OCT scores for identifying plaque, calcium deposition in the wall, and indwelling devices (vascular stents) were significantly higher than those of the IVUS catheter, the scores given to the IVUS images were within the ranking levels that equate to the image quality of IVUS as “very good” and provided sufficiently clear imaging to allow the physician to complete diagnostic review and treatment strategy for PAD.

IVUS imaging has enabled peripheral arterial assessment of vessel luminal dimensions, plaque distribution, morphology, aneurysmal disease, plaque vulnerability, and stent malapposition [16, 17]. Based on similar physical principles of IVUS, intravascular OCT imaging substitutes light waves for ultrasound waves. The usage of near-infrared light in OCT imaging supports subsurface imaging and enables a substantial (10X) increase in resolution over conventional IVUS imaging. The enhanced visualization provided by OCT imaging allows physicians to more clearly interpret and discern among structures in healthy and diseased tissues [18, 19]. However, intravascular OCT imaging is a challenge in vessels of large luminal diameter due to a lower depth of tissue penetration by light rather than sound waves and in the presence of high level of blood that can obscure the light waves, both conditions that are not a challenge for intravascular IVUS. Recently, high-definition IVUS (HD-IVUS) machines have become available for clinical use to not only address anatomical and physiological challenges for OCT imaging but also to provide higher spatial resolution and faster image acquisition than conventional IVUS devices [20]. These HD-IVUS devices have been reported to have significantly better imaging of anatomical and indwelling vascular devices, such as vascular stents, in both bench top and clinical use in comparison to conventional IVUS catheters, but not in comparison to intravascular OCT imaging in the same models [20–22]. While intravascular IVUS imaging has a predominant position in hospitals and office-based laboratories, the characteristics and dimensions of plaque deposition in peripheral artery disease are amenable to OCT imaging due to the smaller luminal dimensions and the necessity of avoiding inadvertent injury to adventitial tissue, which is associated with re-stenosis of peripheral vessels. However, both imaging modalities are important to clinicians to not only assess the condition of the peripheral vessel in order to develop a treatment strategy but also to monitor the progress of the intervention and to identify areas that either require further treatment or that no longer require intervention.

Both IVUS and OCT catheters have the option of being retracted within vessels manually or with the use of a motorized pull-back accessory. A major reason for motorized or automatic pull-back is to facilitate accurate measurement of the length of the area of interest and to capture images that are equidistant within the imaging run [23, 24]. Measuring the length of time that the imaging spanned when set at a specific retraction speed on a motorized accessory can provide the user with an accurate measure of the lesion length in the absence of angiography. However, research has noted that when used in tortuous vessels uniform image capture is not obtained reliably and can cause images to become elongated or foreshortened with the use of a motorized pull-back accessory [25] without improving accuracy against angiography [26]. In this study, both imaging catheters were withdrawn through the vessel manually; however, both physicians had experience with manual retraction of imaging catheters, so the catheters moved through the vessel at comparable speeds with each investigator. In addition, the majority of the images for third-party assessment were captured with the imaging element of each catheter adjacent to the same distance marker on a radiopaque ruler visualized by angiography. With each image used for review and scoring associated with a static mark on the ruler, the variation between the IVUS and the OCT images was minimized. This is demonstrated with the minimal difference in ranking between and among the paired images by the three readers.

Intravascular imaging to support diagnosis and treatment of peripheral artery disease is becoming an important factor in new and evolving endovascular interventions. Use of such imaging can help form the treatment strategy, size the vessel so that the appropriate diameter of balloons or stents being placed is chosen, and guide and direct treatments such as angioplasty and atherectomy to minimize vessel wall injury [6, 27–31]. Intravascular imaging also permits assessment of tissue in the region of the procedure in order to determine whether further treatment is necessary to address regions with incomplete treatment or injury resulting from the treatment [30, 32, 33].

An important variable in the outcome of revascularization procedures is the type and extent of calcium in coronary and peripheral lesions since the incidence of revascularization and treatment success decreases as calcium burden in plaque increases [34–36]. Intravascular imaging to assess calcium in peripheral lesions provides information on the characteristics of calcium present in peripheral lesions as well as burden within the tissue [6, 37–40]. Use of either IVUS or OCT imaging to diagnose and guide treatment of calcified lesions in peripheral vessels has been associated with improved treatment outcomes since they either determine the appropriate path around such deposits or the appropriate treatment method to be used. In this study, diagnosis of calcium within the vessel walls was ranked as close to histology-like for both OCT and IVUS imaging, which would provide the interventionalist with sufficient information to develop treatment options [41]. In this study, both imaging modalities identified the location and extent of calcium in the vessel at levels of clarity that were clinically beneficial.

OCT imaging has a long history in coronary vessels in the assessment of vessel injury following treatment [42–44], and is used in peripheral vessels for not only diagnosis of conditions in the vessels [45] but also has been shown to be capable of characterizing different types of atherosclerotic plaque [14]. In coronary vessel disease, OCT imaging has caused the change in treatment strategy and assessment of the treatment. In the ILUMIEN I study, the information gained from OCT imaging prior to treatment changed the pre-imaging plan in 55% of patients in up to 80% of the cases, primarily in selection of vascular stent length, diameter, and number used [46]. After stent implantation in coronary vessels, OCT imaging is used to assess the stent position and dilatation, which, when corrected, results in better clinical outcomes [46, 47].

OCT imaging in peripheral vessels has been integrated into clinical practice since 2012, with the early results demonstrating assessment of calcium deposition and injury to the vessel wall from previous treatments [41]. With increased use of intravascular OCT imaging, interventionalists are able to review the vessel lumen, wall components, and abnormal physiology in peripheral vessels preoperatively [11, 13–15, 30, 45, 48], which are important conditions to identify in making a diagnosis and choosing procedural options [49]. Intravascular OCT imaging of peripheral vessels containing stents has allowed interventionalists to determine the extent of neointimal tissue growth resulting in in-stent restenosis [31] and the extent of stent revascularization in peripheral vessels following an interventional procedure [31, 33, 50, 51].

OCT-guided atherectomy has clinical benefits of tissue excision with minimal injury to healthy tissue, reduction in the use of contrast agents or radiation, and focused treatment following the identification of plaque distribution and morphology [31]. Schwindt et al. [6] noted that OCT-guided directional atherectomy resulted in 62% of lesions being removed with no disruption or contact with adventitia and 82% of lesions having less than 1% adventitia in excised tissue, which may have contributed to the 92% freedom from target lesion revascularization rate noted at six months post-procedure. Non-radiation imaging such as OCT provides physicians as well as patients with an option of visualization of intravascular conditions with minimal or no contrast or radiation. Contrast use in patients with chronic kidney disease carries risk but use of OCT imaging can be used to measure lesions in lower extremities without use of iodinated contrast agents [49]. OCT imaging provides information on the diameter of peripheral lesions that can direct the physician to choose the correct size of vascular stent or balloon angioplasty, which is associated with longer periods of patency [52]. In contrast, atherectomy relying solely on angiographic guidance can result in suboptimal volumes of plaque excised and injury to the adventitia [30], resulting in restenosis within a shorter period of time than desired [49, 53].

In a similar manner, IVUS imaging prior to and following vascular procedures have been reported to differentiate plaque morphology, assist in stent and balloon sizing, and identification of procedure-related injuries, such as dissection [4, 54, 55], which result in improved clinical outcomes. IVUS-directed percutaneous coronary interventions has been demonstrated to have lower incidence of stent thrombosis [56], while use of IVUS in peripheral vessels can monitor treatment efficacy in real-time, which is associated with lower target lesion revascularization rates up to one year post-procedure [5, 49].

A limitation to this study is the number of subjects enrolled, which may not have provided the breath of physiological and pathological presentations associated with peripheral arterial disease. However, the twelve subjects included in this study did have varying levels of vascular disease, which is more relevant to clinical procedures than previous imaging studies carried out on healthy peripheral vessels.

## Conclusion

From the results of this study, OCT and IVUS imaging are safe and effective methods of examining peripheral vessels in order to diagnose and maximize the clinical benefit of treatment of peripheral artery disease. OCT imaging of peripheral vessels in vivo was comparable to IVUS imaging in the detection of layered structures, treatment zones, and artifacts. OCT was found to be superior to IVUS imaging in the differentiation of plaque, the presence of calcium in the vessel wall, and the presence of vascular stents. Use of intravascular imaging prior to a procedure can direct treatment strategy due to its high resolution of vascular tissue and anomalies in the vessel wall and measuring of lumens to direct choice in appropriate stent sizes. Use of intravascular imaging after an intervention can guide the need for addition intervention such as stent placement or further expansion of poorly implanted stents. Both IVUS and OCT imaging have moved out of the research stage of clinical procedures and into daily use in order to increase the efficacy of vascular interventional procedures without increasing risk.

## Data Availability

The data collected during this study are available from the corresponding author upon reasonable request.

## Abbreviations

IVUS: Intravascular ultrasound
OCT: Optical coherence tomography
PAD: Peripheral artery disease
SFA: Superficial femoral artery
VAS: Visual analogue scale
